# Bedside Approach to Acute Vertigo with Spontaneous Horizontal Nystagmus: The Role of Simultaneous Ice-Water Test Stimulation and Its Correlation with the HINTS Protocol in Differentiating Peripheral and Central Etiologies

**DOI:** 10.3390/audiolres16020039

**Published:** 2026-03-06

**Authors:** Luigi Califano, Cataldo Latorre, Maria Grazia Melillo, Iacopo Cangiano, Giuseppe Manna, Maria Gabriella Coppola, Roberto Teggi

**Affiliations:** 1 Audiology and Phoniatrics Department, AORN San Pio, Via dell’Angelo 1, 82100 Benevento, Italy; luigi.califano1958@gmail.com (L.C.); grazia601@live.com (M.G.M.); 2 Department of Otolaryngology, IRCCS San Raffaele Scientific Institute, San Raffaele Hospital, Via Olgettina 60, 20132 Milan, Italy; cangiano.iacopo@hsr.it (I.C.); teggi.roberto@hsr.it (R.T.); 3 Department of Otolaryngology, San Leonardo Hospital, Viale Europa 79, 80053 Naples, Italy; gmanna13@gmail.com; 4 Emergency Department, AORN San Pio, Via dell’Angelo 1, 82100 Benevento, Italy; mariagabriella.coppola@aornsanpio.it

**Keywords:** acute vertigo acute unilateral vestibulopathy, bedside examination, HINTS, caloric test

## Abstract

Background: Acute vertigo is among the most frequent causes of access to the Emergency Department. In acute vestibular syndrome, differentiating peripheral from central causes remains challenging. The HINTS protocol provides high diagnostic accuracy but requires expertise and adequately informed physicians. The caloric ice-water test has recently been proposed as a bedside tool to aid this differential diagnosis. This study evaluates a novel approach: simultaneous bilateral ice-water irrigation in association with the HINTS protocol. Methods: One hundred consecutive patients presenting with acute vertigo and spontaneous unidirectional nystagmus were enrolled across three Italian centers. All patients underwent clinical assessment including among other the HINTS protocol and the simultaneous bilateral ice-water irrigation. Changes in spontaneous nystagmus during the ice test were recorded. Results: Eighty-six patients fulfilled HINTS criteria for acute unilateral peripheral vestibulopathy, all of whom demonstrated marked suppression/abolition of nystagmus during the simultaneous ice test. In contrast, nystagmus persisted in all 12 patients classified as having central vestibular pathology. Conclusions: Simultaneous bilateral ice-water irrigation is a simple and well-tolerated bedside test that demonstrates strong concordance with the HINTS protocol. While it cannot replace comprehensive clinical assessment, it represents a valuable complementary tool to distinguish peripheral from central causes of acute vertigo.

## 1. Introduction

Since its introduction in the early 1900s, caloric testing has been considered the “gold standard” to identify vestibular loss. It is typically performed with the patient lying supine and the head elevated by 30°, selectively stimulating the horizontal semicircular canal. Warm water induces ampullipetal endolymphatic flow, producing nystagmus toward the irrigated side in a normoreflexic ear. Cold water, in contrast, induces ampullifugal flow, producing contralateral nystagmus [1,2,3]. Very cold water (3–5 °C) has also been used to assess residual vestibular function. In patients with vestibular hypofunction, such strong stimulation typically produces absent or consistently reduced responses [4,5,6].

Acute vertigo and dizziness are among the most common reasons for emergency department visits, accounting for 2.1–4.4% of admissions [7]. Differentiating peripheral vestibular disorders from central nervous system events in patients with recent-onset spontaneous nystagmus remains challenging, since computed tomography (CT) has limited sensitivity for early ischemic lesions [8,9,10]. To address this, the bedside HINTS protocol—Head Impulse Test (HIT), Nystagmus (characteristics), and Test of Skew—has been proposed. HINTS demonstrates a sensitivity of 96–100% and specificity of 96–98%, outperforming MRI within the first 24 h after symptom onset [11,12].

The ice test is also employed in the assessment of brain death in comatose patients [13].

More recently, the ice-water test has been introduced as a simple bedside tool for patients with acute horizontal nystagmus. In peripheral cases, irrigation of the non-pathological ear transiently reduces vestibular function, temporarily attenuating or abolishing nystagmus for 1–2 min. This effect is not observed in central nystagmus [14]. Building on this concept, we explored a novel approach: simultaneous bilateral cold irrigation at 3 °C. We hypothesized that transient bilateral suppression of vestibular responses would eliminate side-to-side asymmetry in patients with acute unilateral vestibular deficit, temporarily suppressing spontaneous horizontal–torsional nystagmus that beats toward the healthy side.

The aim of this study was to evaluate the diagnostic utility of simultaneous bilateral ice-water irrigation and provide initial validation, as this approach has not yet been systematically described in the literature. To this end, the results were compared with established, literature-validated tools—specifically the HINTS protocol and the video Head Impulse Test (vHIT)—which are considered the gold standard for differentiating acute unilateral peripheral from central vestibulopathies.

## 2. Materials and Methods

### 2.1. Study Population and Inclusion Criteria

In this study, we retrospective analyzed recorded data of 100 patients evaluated at three Italian centers for vestibular disorders. All patients were referred from the respective Emergency Departments for acute vertigo between January and June 2025.

Inclusion criteria were as follows:

1. Age > 18 years;
2. Onset of vertigo within the preceding 72 h;
3. No previous history of vertigo of any type;
4. Absence of traumatic events in the previous month;
5. Absence of tympanic perforation;
6. Presence of persistent, long-lasting third-degree nystagmus, unidirectional in all gaze positions, consistently beating in the horizontal plane with a slight torsional component, stationary and sustained—observed for at least 10 min for study purposes;
7. Absence of nystagmus direction changes during diagnostic maneuvers, including the Head Pitching Test, Head Shaking Test, Vibration Test, and positional tests, with the exception of possible inhibition or reversal during the Hyperventilation Test;
8. Absence of involvement of other cranial nerves; and
9. No history of alcohol or drug abuse.

### 2.2. Clinical Assessment

The first step was to perform otoscopy or micro-otoscopy, in order to exclude the presence of tympanic perforation or ear wax.

All patients underwent the HINTS protocol. Specifically:

(a) Nystagmus assessment

Nystagmus was evaluated using binocular infrared videonystagmoscopy (different devices across centers), under both visual fixation and visual deprivation. Nystagmus was classified as peripheral or central according to standard HINTS criteria. Peripheral nystagmus was defined as unidirectional, regular in rhythm and amplitude increasing when gaze was directed toward the fast-phase side, consistent with Alexander’s law. Central nystagmus was suspected when it was direction-changing, predominantly torsional, irregular, or otherwise deviated from expected peripheral patterns, including downhill nystagmus (see below).

(b) Head impulse test [15] was performed according to the well-established patterns: brisk and sudden passive movement of the head, about 20°, high acceleration, in the horizontal plane, fixating a fixed target. The test was considered positive if a centripetal corrective saccade was observed immediately after the impulse, and was negative if it was not.

(c) Test of skew

The alternate cover test was used to detect vertical corrective saccades and to assess the presence of ocular misalignment (hypotropia).

### 2.3. Simultaneous Ice Test

All patients underwent the simultaneous ice test at the end of the clinical evaluation, specifically after HIT and video-HIT, to avoid false results due to inhibition of both lateral canals. Both ears were irrigated simultaneously with 10 mL of water at approximately 3 °C while the patient was supine with the head elevated 30°. This technique does not require a caloric irrigator. A single individual can perform it, since the test does not need the same precision as in caloric testing. Before the irrigation, an otoscopy was performed to exclude the presence of cerumen; if present, it was removed through suction or with a hook. Instead, four ice cubes are placed in about 10 mL of water and left to equilibrate for several minutes. A standard thermometer was used to measure the temperature during this phase, until the desired value was reached. Changes in nystagmus frequency were recorded via videonystagmoscopy under visual deprivation. The test was classified as positive if it induced complete or near-complete inhibition of nystagmus, and negative if spontaneous nystagmus persisted. Results of the ice test were subsequently compared with those of the HINTS protocol and vHIT to establish whether suspected central involvement was consistently identified across modalities.

### 2.4. Audiological Testing

This included pure-tone audiometry (125–8000 Hz) and, when clinically indicated, impedance audiometry. The examination was performed within 5 days of acute onset, or immediately if new hearing complaints were reported.

### 2.5. Video Head Impulse Test

vHIT was performed according to established standards. Pathological vestibulo-ocular reflex (VOR) gain was defined as <0.80 for the horizontal canals and <0.70 for the vertical canals, accompanied by corrective saccades. Both HIMP and SHIMP protocols were assessed. The results were also classified qualitatively as either presence or absence of corrective saccades. The test was performed using a commercially available system (ICS Impulse, Otometrics, Taastrup, Denmark).

## 3. Results

The study cohort comprised 100 subjects (51 males and 49 females), with a mean age of 54.03 ± 15.12 years. All patients were evaluated between 1 and 3 days after the onset of vertigo. At presentation, all subjects exhibited third-degree unilateral spontaneous nystagmus, partially suppressed by visual fixation and without directional changes, in accordance with the Bárány Society diagnostic criteria for acute unilateral vestibulopathy (AUVP) [16].

### 3.1. HINTS Examination

HINTS was performed in all patients. In 86 patients, the HINTS protocol fulfilled diagnostic criteria. Findings included unidirectional third-degree nystagmus, a negative test of skew and a positive video-HIT with a reduced VOR gain with the presence of OVERT saccades.

In two patients—a 38-year-old woman and a 19-year-old man—HINTS criteria were not completely fulfilled. Both exhibited spontaneous, third-degree, arrhythmic nystagmus. They presented a normal VOR gain, but with the presence of corrective saccades turning the head on one side.

In the remaining 12 patients, HINTS criteria were not fulfilled. Findings included third-degree horizontal, arrhythmic nystagmus with absent or reduced visual suppression and high-grade ocular hypotropia in four cases. The vHIT showed normal VOR gain and corrective saccades were absent. Several patients exhibited more than one of these findings. The final diagnoses of these subjects was performed in the days following the first examination and is reported in the Table 1. Seven of them presented microischemic lesions without signs of recent bleeding; it can be postulated that the pathophysiological mechanism may be linked to an acute lesion in the posterior fossa not identified by MRI for the delay of execution of the exam.

Overall, HINTS criteria were fulfilled in 86 patients and not fulfilled—either partially or entirely—in the remaining 14 patients, who were therefore classified as having a central etiology. The central group (5 females, 9 males) was significantly older than the peripheral group, with a mean age of 61.1 ± 9.87 years versus 51.6 ± 14.7 years, respectively (t = 3.1, two-tailed *p* = 0.005).

### 3.2. Simultaneous Ice Test

This test was performed in all patients. Nystagmus was markedly suppressed (*n* = 11) or completely abolished (*n* = 77) in 88 patients. This group included the 86 individuals who fulfilled HINTS diagnostic criteria for AUVP, as well as the two patients who did not completely fulfill the HINTS criteria since the VOR gain was within limits, but demonstrated corrective saccades on the video head impulse test. In 72 of the 86 cases who fulfilled HINTS criteria, suppression of nystagmus was accompanied by the emergence of a transient vertical upbeat nystagmus. Notably, this phenomenon was observed in 72 of 74 patients with lateral and anterior canal deficits but with normal posterior canal function, as demonstrated by vHIT. Patients with inhibition of nystagmus reported a significative decrease of subjective vertigo.

In 12 patients, the simultaneous ice test did not inhibit spontaneous nystagmus. Interpretation of the ice test in isolation, without integration with the HINTS protocol, would have classified 88 patients as peripheral and 12 patients as central.

In summary, all patients who did not fulfill the HINTS protocol also showed no inhibition of nystagmus during the simultaneous ice test, with the exception of two cases.

All clinical results, including HINTs criteria, simultaneous ice test, and HIT/v-HIT, are summarized in Table 1.

### 3.3. Audiological Testing

This test was performed in 90 patients: 82 in the peripheral group and 8 in the central group. Five patients in the peripheral group reported the onset of hearing symptoms associated with acute vertigo. In two of these cases, severe pantonal hearing loss was observed on the same side as the vestibular deficit, while the remaining three cases exhibited high-frequency hearing loss. No relevant findings were observed in the other patients. Notably, the test could not be performed in the woman who partially fulfilled HINTS criteria due to her serious clinical condition.

### 3.4. Imaging

The MRI in the two patients (a 39-year-old woman and a 19-year-old man) presenting normal VOR gain but corrective saccades and inhibition of nystagmus with the simultaneous ice test showed, respectively, a cerebellar infarct, involving the left AICA territory and the right PICA territory, and demyelinating lesions in the brainstem near the emergence of the eighth cranial nerve and in the superior and middle cerebellar peduncles.

The clinical results of these two “exceptions” are summarized in Table 2.

Among the 14 patients classified as central based on integration of all tests, confirmed central lesions on CT and/or MRI were observed in five patients: a 60-year-old man and a 35-year-old woman with cerebellar neoplasms; a 75-year-old man with pontine ischemia; an 82-year-old woman with Wallenberg syndrome; and a 24-year-old woman experiencing the first episode of multiple sclerosis, with active cerebellar and brainstem lesions. In this group, HINTS criteria were not fulfilled, and the simultaneous ice test failed to inhibit nystagmus.

In the remaining seven patients, no specific imaging diagnosis was obtained; most showed only nonspecific leukoaraiosis or old, non-active vascular lesions. It can be hypothesized that since MRI was performed some days after the acute vertigo, a small bleed may be difficult to be identified in the posterior fossa.

## 4. Discussion

HINTS has demonstrated reliability in differentiating peripheral from central causes of vertigo during their sudden onset, since a CT scan is often unable to detect a cerebellar or brainstem ischemic lesion [16]. The problem is of remarkable significance in emergency departments, since acute vertigo is one of the most important causes of access [17,18]. To be considered as peripheral, vertigo should fulfill all clinical signs of the HINTS: nystagmus should be horizontal, in many cases with a torsional component, it should increase when the patient rolls the eyes toward the side in which the nystagmus is beating (Alexander’s law), it must have at least a partial inhibition, and a cover test must be negative. All these clinical signs are, in our opinion, easy to discern using simple Frenzel glasses. Moreover, a bedside HIT must show corrective saccades when rotating the head toward the pathological side [10]. In our opinion, this maneuver has a learning curve; it may be easy and reliable for more expert examiners but it can create some doubts in less experienced clinicians. The high turnover of physicians in our largest hospitals should also be considered, which often does not permit physicians to properly learn the methodology. In the last years, several video assisted systems have been proposed to perform the exam, which can test the function of all six canals; however, this is not widely available in an emergency room. A recent Cochrane review established that clinical HINTS examination was 94.0% sensitive in detecting a central disorder, while the sensitivity of the video-assisted HINTS examination in different studies ranges from 85.0% to 100%; they also underline that the HINTS examination had good sensitivity to diagnose a central cause for AVS in the emergency department, especially when performed by trained clinicians [19].

Caloric testing has been widely used to assess the function of each labyrinth; when the patient is lying with the head raised by 30°, only the lateral canal is stimulated. Warm water provokes a movement of the endolymph toward the cupola, thus provoking a nystagmus beating on the same side; the opposite occurs when using cold water, which inhibits lateral canal function [20]. In particular, the ice test, which is performed with water near 0°, has been studied in patients with a unilateral weakness at caloric tests [6] and more recently has been proposed in patients with a spontaneous nystagmus to differentiate peripheral from central vestibular disorders [12].

Inhibition of both lateral canals is expected from the simultaneous stimulation of both ears with water near 0°. In a patient with an acute onset of asymmetric peripheral vestibular function and a spontaneous nystagmus, the ice test will provoke an inhibition of the best functioning side, while less or no inhibition on the ear with peripheral vestibular loss, leading to a significant reduction/disappearance of spontaneous nystagmus. In contrast, in the case of a central nystagmus, inhibition of the lateral canals will not provoke changes in the spontaneous nystagmus.

As far as we know, this is the first study to compare the results of HINTS with the ice test. Of note, the 86 subjects fulfilling HINTS criteria also presented inhibition of nystagmus with the ice test. Two patients presented a VOR gain within normal limits but with corrective saccades; they also presented a reduction of nystagmus during the ice test. It could be speculated that these patients presented a disorder provoked by both peripheral and central factors. In the woman, a peripheral damage could have preceded the cerebellar infarct, while in the male subject a lesion of the root entry of the eighth cranial nerve was the reason for the partial deficit. This finding nonetheless underlines that the ice test may be a useful bedside tool to exclude a central disorder, but as it could present potential limitations, it alone cannot replace the HINTS protocol or other tests but should be considered a complementary exam assisting in the differential diagnosis process.

## 5. Conclusions

The ice test is a rapid, simple, and easily interpretable bedside examination. It shows a high level of agreement with the HINTS protocol and represents a reliable complementary diagnostic tool in the emergency department, particularly in cases with inconclusive HINTS findings. The ice test may also be especially valuable for otolaryngologists and other clinicians with limited experience or confidence in performing the head impulse test.

A future goal of this test might be to detect the minimum stimulus necessary to provoke a decrease/inhibition of nystagmus also in patients with different vestibular disorders, including vestibular migraine and Menière’s disease, both in acute vertigo and in patients with smooth spontaneous nystagmus detected only with infrared googles. It can be speculated that it can be useful to assess if pathophysiology of the finding may be peripheral or central,

## Figures and Tables

**Table 1:** HINTs criteria, simultaneous ice test nystagmus responses, HIT/v-HIT and imaging finding of the total sample.

Patient Group	Sample	HINTS Criteria	Simultaneous Ice Test	HIT/v-HIT Findings	Imaging Findings
Peripheral (AUVP)	86	Fulfilled	Suppressed/abolished nystagmus (*n* = 86)	Centripetal corrective saccades; lateral/anterior canal deficits	No central lesions
Central (partially fulfilled)	2	Partially fulfilled	Suppressed nystagmus	Corrective saccades present	1 double cerebellar infarct (AICA L, PICA R); 2 demyelinating lesions in brainstem & superior/middle cerebellar peduncles
Central (not fulfilled)	12	Not fulfilled	Not inhibited nystagmus	Corrective saccades absent	5 confirmed central lesions; 2 cerebellar neoplasms; 1 pontine ischemia; 1 Wallenberg syndrome; 1 first MS episode; 7 nonspecific leukoaraiosis/old vascular lesions also in posterior fossa

**Table 2:** HINTs criteria, simultaneous ice test nystagmus responses, HIT/v-HIT and imaging finding of the two “exceptions”.

Patient Age/Sex	HINTS Criteria	Simultaneous Ice Test	HIT/v-HIT Findings	Imaging Findings
39 F	Not fulfilled	Suppressed nystagmus	Corrective saccades present	Double cerebellar infarct: left AICA, right PICA
19 M	Not fulfilled	Suppressed nystagmus	Corrective saccades present	Demyelinating lesions in brainstem & superior/middle cerebellar peduncles

## Data Availability

The data supporting the presented results of the study are available on request from the authors.

## References

[1] Lorente de No R., Graham M.D., Kemink J.L. (1987). Facets of the life and work of Professor Robert Bárány (1886–1936). The Vestibular System: Neurophysiologic and Clinical Research.

[2] Hood J.D. (1989). Evidence of direct thermal action upon the vestibular receptors in the caloric test. Acta Otolaryngol..

[3] Scherer H., Brandt U., Clarke A.H., Merbold U., Parker R. (1986). European vestibular experiments on the Spacelab-1 mission: 3 Caloric nystagmus in microgravity. Exp. Brain Res..

[4] Barber H.O., Stockwell C.W. (1980). Manual of Electronystagmography.

[5] Jacobson G.P., Shepard N.T. (2008). Balance Function Assessment and Management.

[6] Batuecas-Caletrio A., Montes-Jovellar L., Boleas-Aguirre M.S., Perez-Fernandez N. (2009). The ice-water caloric test. Acta Otolaryngol..

[7] Newman-Toker D.E., Edlow J.A. (2015). TiTrATE: A Novel, Evidence-Based Approach to Diagnosing Acute Dizziness and Vertigo. Neurol. Clin..

[8] Lee S.U., Tarnutzer A.A. (2025). Usefulness of Nystagmus Patterns in Distinguishing Peripheral From Central Acute Vestibular Syndromes at the Bedside: A Critical Review. J. Clin. Neurol..

[9] Newman-Toker D.E., Cannon L.M., Stofferahn M.E., Rothman R.E., Hsieh Y.-H., Zee D.S. (2007). Imprecision in patient reports of dizziness symptom quality: A cross-sectional study conducted in an acute care setting. Mayo Clin. Proc..

[10] Kattah J.C., Talkad A.V., Wang D.Z., Hsieh Y.H., Newman-Toker D.E. (2009). HINTS to diagnose stroke in the acute vestibular syndrome: Three-step bedside oculomotor examination more sensitive than early MRI diffusion-weighted imaging. Stroke.

[11] Kattah J.C. (2018). Use of HINTS in the acute vestibular syndrome. An Overview. Stroke Vasc. Neurol..

[12] Gufoni M., Casani A.P. (2023). The role of vestibular cold caloric tests in the presence of spontaneous nystagmus. Acta Otorhinolaryngol. Ital..

[13] Greer D.M., Kirschen M.P., Lewis A., Gronseth G.S., Rae-Grant A., Ashwal S., Babu M.A., Bauer D.F., Billinghurst L., Corey A. (2023). Pediatric and Adult Brain Death/Death by Neurologic Criteria Consensus Guideline. Neurology.

[14] Strupp M., Bisdorff A., Furman J., Hornibrook J., Jahn K., Maire R., Newman-Toker D., Magnusson M. (2022). Acute unilateral vestibulopathy/vestibular neuritis: Diagnostic criteria. J. Vestib. Res..

[15] Halmagyi G.M., Curthoys I.S. (1988). A clinical sign of canal paresis. Arch. Neurol..

[16] Newman-Toker D.E. (2016). Missed stroke in acute vertigo and dizziness: It is time for action, not debate. Ann. Neurol..

[17] Tarnutzer A.A., Berkowitz A.L., Robinson K.A., Hsieh Y.-H., Newman-Toker D.E. (2011). Does my dizzy patient have a stroke? A systematic review of bedside diagnosis in acute vestibular syndrome. Can. Med. Assoc. J..

[18] Spencer R., Gandhi J., Tepe J., Li C., Kulzer M., O’nEill J., Eisenmenger L., Goldberg M., Chien A., Chang W. (2025). Low incidence of acute actionable imaging findings in emergency department patients imaged for vertigo: Retrospective analysis and proposed guidelines. Emerg Radiol..

[19] Gottlieb M., Peksa G.D., Carlson J.N. (2023). Head impulse, nystagmus, and test of skew examination for diagnosing central causes of acute vestibular syndrome. Cochrane Database Syst Rev..

[20] Gonçalves D.U., Felipe L., Lima T.M. (2008). Interpretation and use of caloric testing. Braz. J. Otorhinolaryngol..

